# Comparison of novel markers of metabolic complications and cardiovascular risk factors between obese non-diabetic and obese type 1 diabetic children and young adults

**DOI:** 10.3389/fendo.2022.1036109

**Published:** 2022-12-12

**Authors:** Anna Kącka, Anna Charemska, Elżbieta Jarocka-Cyrta, Barbara Głowińska-Olszewska

**Affiliations:** ^1^ Department of Clinical Pediatrics, Faculty of Medical Sciences, University of Warmia and Mazury in Olsztyn, Provincial Specialist Children’s Hospital, Olsztyn, Poland; ^2^ Department of Pediatrics, Endocrinology, Diabetology with Cardiology Division, Medical University of Białystok, Białystok, Poland

**Keywords:** type 1 diabetes mellitus, obesity, fetuin-A, E-selectin, osteoprotegerin

## Abstract

**Introduction:**

For the past years, the prevalence of obesity is growing in the general population of children, as well as among diabetic patients, resulting in increased risk of cardiovascular complications. Type 1 diabetes mellitus (T1DM) is one of the most common chronic diseases in children and young adults, leading to decreased life quality and lifespan, with obesity being recognized recently as a major contributing factor to these health problems. The objective of this study was to analyze and compare the selected novel markers for metabolic complications of obesity and vascular risk factors between obese non-diabetic and obese T1DM children and young adults.

**Methods:**

One hundred four subjects, aged between 10 and 24 years (31 with T1DM and excessive body weight, 41 with obesity without diabetes, and 32 with T1DM and normal weight), and 32 matched lean controls were included in the study. Clinical characteristics, blood pressure measurements, daily requirement for insulin, HbA1c%, plasma lipids, fetuin-A, E-selectin, and osteoprotegerin levels were compared with respect to body mass index (BMI), body mass index standard deviation score (BMI-SDS), and carotid intima-media thickness (cIMT) of common carotid arteries.

**Results:**

Patients with T1DM and excessive body weight compared to non-diabetic obese subjects had similar values of systolic blood pressure (125.6 ± 8.2 vs. 127.3 ± 12.9 mmHg, *p* = 0.515), diastolic blood pressure (78.19 ± 7.03 vs. 78.02 ± 8.01 mmHg, *p* = 0.918), cholesterol (175.26 ± 34.1 vs. 163.51 ± 26.08 mg/dl, *p* = 0.102), LDL (108.03 ± 32.55 vs. 112.22 ± 26.36 mg/dl, *p* = 0.548), and triglyceride levels (118.19 ± 71.20 vs. 117 ± 55.80 mg/dl, *p* = 0.937); all values were found to be higher compared to non-obese T1DM and healthy controls. HbA1c level and insulin resistance indices were significantly worse in T1DM obese vs. T1DM non-obese patients. Fetuin-A levels were higher among obese non-diabetic patients (*p* = 0.01), and E-selectin and osteoprotegerin levels were similar in both groups with obesity, but higher than in the reference group. There were no statistical differences in cIMT with T1DM with normal weight, excessive weight, and non-diabetic obese children; however, the cIMT value was higher compared to the reference group.

**Discussion:**

Novel markers of metabolic complications of obesity are similar between obese T1DM and non-diabetic subjects. Obesity in patients with T1DM results in worse metabolic control, insulin resistance, and increased risk for vascular complications.

## Introduction

In the last decades, the prevalence of obesity and diabetes mellitus has been rapidly growing (1). Once considered a problem in high-income countries, overweight and obesity are nowadays recognized as an epidemic worldwide, resulting in metabolic complications such as insulin resistance, dyslipidemia, hypertension, metabolic syndrome, and non-alcoholic fatty liver disease. It is also recognized as a risk factor for cardiovascular diseases.

According to WHO, over 39 million children under 5 years and 340 million children aged 5–19 are overweight or obese (2–4). Type 1 diabetes mellitus (T1DM) is one of the most common chronic diseases leading to decreased life quality and lifespan. The main cause of death among diabetic patients is cardiovascular complications. T1DM itself is recognized as a high-risk factor for cardiovascular disease (5–7). Others include hypertension, decreased HDL, and increased triglyceride levels. The prevalence of obesity in patients with T1DM is also rising and is associated with insulin pump therapy, early onset, puberty, female sex, and low levels of physical activity. Excessive body weight in children with T1DM has recently been recognized as a significant factor contributing to complications on various stages of the disease. Furthermore, the prevalence of insulin resistance, associated with being overweight and/obese, was previously linked to type 2 diabetes and is increased in children with T1DM (8). It is now recognized that insulin resistance results in worse glycemic control, higher HbA1c, and higher atherogenic lipid profile, and contributes to earlier development of microangiopathy as well as macroangiopathy. Measurement of insulin resistance in T1DM is difficult due to hypoinsulinemia, and methods such as HOMA-IR (homeostasis model assessment of insulin resistance) cannot be used. Therefore, the euglycemic–hyperinsulinemic clamp has been proposed, but not commonly used in practice, because it is labor-intensive and invasive. For clinical purposes, estimated glucose disposal rate (eGDR) has been developed, which is strongly correlated with clamp-measured insulin resistance. The formula is based on clinical measurements, such as hypertension status, waist-to-hip ratio, and HbA1c%, or especially in children—age, daily insulin requirement, and HbA1c% (9–12).

Additionally, to estimate the visceral adiposity dysfunction associated with cardiometabolic risk, a sex-specific index based on waist circumference, BMI, triglycerides, and HDL—visceral adiposity index (VAI)—was created (13).

The obesity epidemic caused increased interest in factors released by adipose tissue, such as inflammatory cytokines, fatty acids, and adipocytokines. In addition to well-described adipocytokines and markers of the inflammatory process, new prognostic indicators of an increased risk of developing cardiovascular diseases are still being sought.

While recent studies have shown a good correlation between risk of cardiovascular disease and some novel metabolic markers [osteoprotegerin (OPG), fetuin-A, and E-selectin] in adults, limited studies have been conducted in children (14).

Fetuin-A (Alpha-2 Heremans Schmid glycoprotein), which is a negative acute phase, also causes insulin resistance by enhancing insulin receptor tyrosine kinase activity and insulin receptor auto-phosphorylation. The fetuin-A production is increased by hyperlipidemia and hyperglycemia. The current studies provide evidence that higher levels of fetuin-A are also associated with higher risk of cardiovascular complications (15). In a case–cohort study, Weikert et al. showed that patients with high fetuin-A concentrations had a fourfold increased risk for myocardial infarction and ischemic stroke compared to subjects with low fetuin-A levels (16). OPG is a cytokine member of the tumor necrosis factor (TNF) involved in bone metabolism and vascular calcification and atherogenesis (17). Recent studies showed that the RANK/RANKL/OPG pathway is important for the regulation of obesity, as well as associations between OPG levels and ischemic heart disease and insulin resistance (18). Alharbi et al. found that serum OPG level was significantly elevated in obese with insulin resistance patients compared to control subjects (19).

Moreover, Perez de Ciriza et al. in their study showed that patients with the metabolic syndrome had higher OPG than patients without. OPG correlated with carotid intima-media thickness (cIMT) and patients with atherosclerosis had higher OPG concentrations (20).

E-selectin is an endothelial adhesion molecule known to be integrally involved in the development of atherosclerotic plaque by promoting the adhesion of leukocytes to the endothelial wall. Levels of E-selectin are also increased in obesity (21). The MIAMI study that examined the relationship between various circulating markers of inflammation and CIMT found that E-selectin was strongly correlated to atherosclerotic burden and CIMT and inversely correlated to HDL-c (22).

Recent studies have shown that obesity and T1DM in youth are associated with greater cIMT. It is also influenced by hypertension, dyslipidemia, and poor glycemic control (23–25).

The objective of the study was to analyze and compare selected novel markers of metabolic complications of excessive body weight and classical cardiovascular risk factors between obese non-diabetic and obese T1DM children. The following variables were analyzed: daily insulin requirement, insulin resistance, measurement of the cIMT, and levels of fetuin-A, E-selectin, and OPG.

Therefore, the aim of this study was to investigate the link between obesity among type 1 diabetic patients, novel markers, and risk of cardiovascular complications.

We hypothesized that our results might help to identify the group of patients with higher risk of macroangiopathy as well as create the therapeutic goals for these patients, which might delay the development of chronic complications.

## Methods

The study was performed in the Pediatric Endocrinology and Diabetology Division, Department of Clinical Pediatrics as well as Outpatient Clinics of Provincial Specialist Children’s Hospital in Olsztyn between 2019 and 2022. The Ethics Committee of University of Warmia and Mazury approved this study (approval number KB/13/2019). Written informed consent forms were acquired from parents and patients older than 16 years.

### Patients

One hundred four patients, aged between 10 and 24 years (31 with T1DM and obesity, 41 with obesity, and 32 with T1DM and normal weight), were enrolled in the study. The onset of T1DM must have been at least 2 years prior. The control group consisted of 32 age-matched healthy peers (BMI < 90 pc and BMI-SDS < 1).

The following inclusion criteria were used for the participants: excessive body weight was defined by BMI > 90 pc and BMI-SDS > 1 for children with T1DM and BMI > 97 pc and BMI-SDS > 2 for children with simple obesity based on BMI-for-age percentile charts of the nationally representative group. Clinical remission of diabetes, more than one autoimmune comorbidity, and microvascular complications were exclusion criteria for children with T1DM. Further exclusion criteria for all the participants included acute infection, previous surgery, or trauma 1 month prior.

### Physical examination and clinical data

Weight, height, and waist circumference were measured. Body mass index (BMI kg/m^2^) was calculated by the following formula: weight (kg)/height^2^ (m^2^). Standardized BMI (BMI-SDS) was calculated by the following formula: (BMI − BMI 50 pc)/0.5× (BMI 50 pc − BMI 3 pc). Waist circumference SDS was calculated by the following formula: (waist circumference − waist circumference 50 pc)/0.5× (waist circumference 50 pc − waist circumference 3 pc). Obtained data were referenced to polish percentile charts according to age and sex (26). The average of three measurements was taken to determine blood pressure.

In patients with T1D, data including diabetes duration and daily requirement of insulin were collected.

### Laboratory methods

Venous blood samples were obtained after 8–12 h of fasting for laboratory tests. Eight milliliters of blood was collected and then centrifuged for 10 min at 2,000 turns per minute. Blood tests, including glycated hemoglobin (HbA1c), total cholesterol (TC), low-density lipoprotein (LDL), high-density lipoprotein (HDL), blood glucose (BG), insulin, alanine aminotransferase (ALT), aspartate aminotransferase (AST), gamma-glutamyl transferase (GGT), and 25-hydroksyvitamin D, were performed using standard methods in the Diagnostic Laboratory of Provincial Specialist Children’s Hospital in Olsztyn. The remaining material (serum) was stored at a temperature of −80°C until the determination.

To analyze novel markers of metabolic complications and cardiovascular risk factors including fetuin-A, E-selectin, and OPG, commercially accessible rapid sandwich immunoassay ELISA kits were used at the Institute of Animal Reproduction and Food Research of Polish Academy of Science.

In addition, in children with obesity, oral glucose toleration test (OGTT) was performed (1.75 g/kg, maximum 75 g of glucose). Insulin sensitivity was estimated by the homeostasis model assessment of insulin resistance (HOMA-IR) index using the following formula: fasting insulin × fasting blood glucose (mg/dl)/405. Interpretation of OGTT and diagnosis of prediabetes were established according to the criteria of Polish Society of Diabetes 2022 (27).

The following formulas were used for assessment of insulin resistance: for non-diabetic patients, HOMA-IR, while for type 1 diabetic patients, eGDR.

Additionally, visceral adipose function was expressed by the VAI.

eGDR was calculated using two indirect methods:

eGDR 1: 20.91 + [1.51 × (boy 1, girl 0)] − [0.1 × (age in years)] − [0.13 × (waist circumference in cm)] − [0.3 × HbA1c%] − [2.11 × daily insulin requirement], and eGDR2: 21.158 + (−0.09 × waist circumference in cm) + (−3.407 × 1 for HBP) + (−0.551 × HbA1c%). Lower values indicate greater insulin resistance (28, 29).

VAI was calculated according to sex using the following formula: girls: (waist circumference/36.58 + [1.89 × BMI]) × (TG/0.81) × (1.52/HDL) boys: (waist circumference/39.68 + [1.88 × BMI]) × (TG/1.03) × (1.31/HDL) (13).

### Ultrasound

PHILIPS, Toshiba Apolio 500 ultrasound devices were used for measuring cIMT based on standard protocol. Covered end-diastolic (minimum diameter) IMT of the far walls (the distance between the leading edge of the first echogenic line and the leading edge of the second echogenic line) within a distance larger than 1 cm from the bifurcation was measured. The mean value of six measurements (three from the left and three from the right carotid artery) was included in the analyses (30, 31).

### Statistical analysis

Statistical analysis was performed using STATISTICA v.13.3 software. Quantitative variables were expressed as mean and standard deviation (SD).

The values of categorized variables were presented in terms of cardinality (*N*).

To analyze the differences between the studied parameters in individual groups, the following parametric tests were used: Student’s *t*-test for the comparison of two groups and the ANOVA test (with the *post-hoc* NIR test) in the case of a larger number of groups for the variables expressed on the quantitative scale.

The analysis of correlations was performed using the Pearson correlation coefficient.

Statistically significant results were found at the level of *p* < 0.05.

## Results

Patients with T1DM and excessive body weight compared to non-diabetic obese subjects had similar values of systolic blood pressure (125.6 ± 8.2 vs. 127.3 ± 12.9 mmHg, *p* = 0.515), diastolic blood pressure (78.19 ± 7.03 vs. 78.02 ± 8.01 mmHg, *p* = 0.918), cholesterol (175.26 ± 34.1 vs. 163.51 ± 26.08 mg/dl, *p* = 0.102), LDL (108.03 ± 32.55 vs. 112.22 ± 26.36 mg/dl, *p* = 0.548), and triglyceride levels (118.19 ± 71.20 vs. 117 ± 55.80 mg/dl, *p* = 0.937); all values were higher compared to non-obese T1DM and healthy controls. The general characteristics of the study groups are shown in **Table 1**.

**Table 1:** General characteristics of the study groups.

	Obese group (*N* = 41)	T1DM group (*N* = 32)	T1DM obese group (*N* = 31)	Control group (*N* = 32)	*p*
Gender (M/F) [*n* (%)]	16 (39.0%)/25 (61.0%)	13 (40.6%)/19 (59.4%)	10 (32%)/21 (68%)	9 (28.1%)/23 (71.9%)	
Age [years]	13.82 ± 2.87 ^bc^	15.19 ± 3.29	15.63 ± 2.59 ^a^	13.84 ± 2.70	0.017
Height [cm]	164.34 ± 11.59	163.94 ± 12.00	164.72 ± 11.42	160.15 ± 13.30	0.395
Body weight [kg]	92.29 ± 23.84 ^abc^	54.44 ± 13.05 ^b^	73.45 ± 12.98 ^a^	48.88 ± 10.88	0.0001
BMI [kg/m^2^]	33.82 ± 6.32 ^abc^	20.00 ± 2.55 ^b^	26.85 ± 2.53 ^a^	18.70 ± 2.31	0.0001
BMI-SDS	5.24 ± 2.20 ^abc^	0.21 ± 0.64 ^b^	2.49 ± 0.87 ^a^	−0.05 ± 0.59	0.0001
Waist circumference [cm]	97.87 ± 13.66 ^abc^	69.00 ± 7.27 ^ab^	80.42 ± 6.98 ^a^	63.86 ± 6.27	0.0001
Waist circumference—SDS	4.62 ± 1.93 ^abc^	0.25 ± 0.65 ^b^	1.83 ± 0.80 ^a^	−0.21 ± 0.66	0.0001
Systolic blood pressure [mm/Hg]	127.32 ± 12.93 ^ac^	120.16 ± 10.02 ^ab^	125.58 ± 8.18 ^a^	110.44 ± 7.10	0.0001
Diastolic blood pressure [mm/Hg]	78.01 ± 8.01 ^ac^	73.94 ± 7.95 ^ab^	78.19 ± 7.03 ^a^	69.88 ± 7.30	0.0001
Cholesterol [mg/dl]	163.51 ± 26.08 ^a^	164.69 ± 32.42 ^a^	175.26 ± 34.10 ^a^	149.06 ± 26.25	0.007
TG [mg/dl]	117.00 ± 55.80 ^a^	71.31 ± 18.76 ^b^	118.19 ± 71.2 ^a^	63.03 ± 27.42	0.0001
HDL [mg/dl]	48.22 ± 9.25 ^abc^	62.09 ± 12.30	59.97 ± 15.76	58.56 ± 14.31	0.0001
LDL [mg/dl]	112.22 ± 26.36 ^a^	99.75 ± 28.72 ^a^	108.03 ± 32.55 ^a^	85.91 ± 20.66	0.001

The data are presented as mean ± SD; ANOVA test.

a p < 0.05, compared to the control group.

b p < 0.05, compared to T1DM obese.

c p < 0.05, compared to T1DM in post-hoc tests.

We noted a difference in glycemic control among diabetic patients. Mean HbA1c levels from the year prior to the study and obtained during the study were higher in obese T1DM patients, 7.99 ± 0.93% and 8.1 ± 1.21%, than in non-obese T1DM patients, 7.61 ± 0.89% and 7.780 ± 0.91% (*p* = 0.099, *p* = 0.252), although not significant. Daily dosage of the insulin was similar; however, insulin resistance indices eGDR1 and eGDR2 were significantly lower in obese T1DM patients than in non-obese T1DM patients: eGDR1: 5.16 ± 1.33 vs. 6.96 ± 1.32; eGDR2: 9.37 ± 1.21 vs. 10.66 ± 0.9 (*p* = 0.0001, *p* = 0.0001), meaning insulin resistance (**Table 2**). Daily dosage of the insulin was similar 0.83±0.16 in T1DM vs 0.85±0.17 [IU/kg/24hrs] (p=0.593).

**Table 2:** Glycemic control in type 1 diabetic patients .

	T1DM group	T1DM obese group	p
Gender M/F(%)	13 (40,6%)/	10 (32,3%)/	
	19 (59.4%)	21 (67,7%)	
Age [years]	15.19±3.29	15.63±2.59	0.56
	
Height [cm]	163.94±12.00	164.72±11.42	0.791
	
Body weight [kg]	54.44±13.05	73.45±12.98	0.0001*
	
T1D duration [years]	8.67±4.52	7.09±2.96	0.107
	
Mean HbA1c from last year	7.61±0.89	7.99±0.93	0.099
	
HbA1c last [%]	7.78±0.91	8.10±1.21	0.252
	
Daily insulin requirement [IU/kg/24hrs]	0.83±0.16	0.85±0.17	0.593
	
eGDR1	6.96±1.32	5.16±1.33	0.0001*
	
eGDR2	10.66±0.90	9.37±1.21	0.0001*
	

The data are presented as mean±SD *p<0.05 in t-student test.

**Figure 1:**
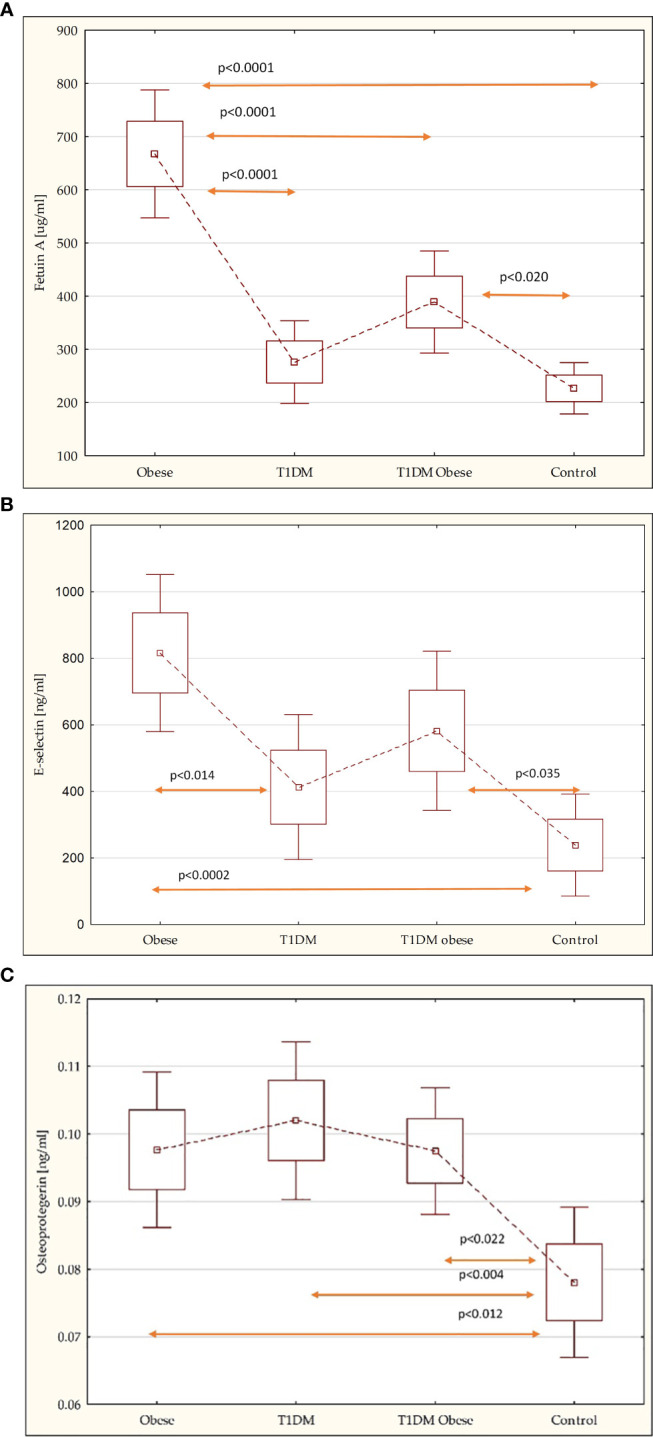
**(A)** Comparison of the fetuin-A in the study groups. **(B)** Comparison of the E-selectin in the study groups. **(C)** Comparison of the osteoprotegerin in the study groups.

Comparing novel markers of metabolic complications and cardiovascular risk factors revealed that fetuin-A levels were higher among obese non-diabetic children, 667.18 ± 363.12 vs. 388.87 ± 253.75 [μg/ml] (*p* = 0.01); E-selectin, 815.87 ± 751.92 vs. 582.01 ± 645.75 [ng/ml], and OPG levels, 0.10 ± 0.04 vs. 0.10 ± 0.03 [ng/ml], were similar in both groups with obesity. However, there was statistical difference in E-selectin and OPG levels between the obese non-diabetic group, the obese with T1DM group, and the control group: 238.78 ± 434.90 [ng/ml] (*p* < 0.0002; *p* < 0.035); 0.08 ± 0.03 [ng/ml] (*p* < 0.012; *p* < 0.022) (**Table 3**; **Figures 1A–C**).

**Table 3:** Comparison of the novel markers between non – diabetic and T1DM obese patients.

	Obese group		T1DM obese group		p
	Mean±SD	Med	Mean±SD	Med	
E-selectin [ng/ml]	815.87±751.92	296.78	582.01 ± 645.75	274.13	0.280
Osteoprotegerin [ng/ml]	0.10 ± 0.04	0.10	0.10 ± 0.03	0.09	0.914
Fetuin A [ug/ml]	667.18 ± 363.12	807.42	388.87 ±253.75	300.26	0.01*

The data are presented as mean±SD.

*p < 0.05 in t-student test.

**Figure 2:**
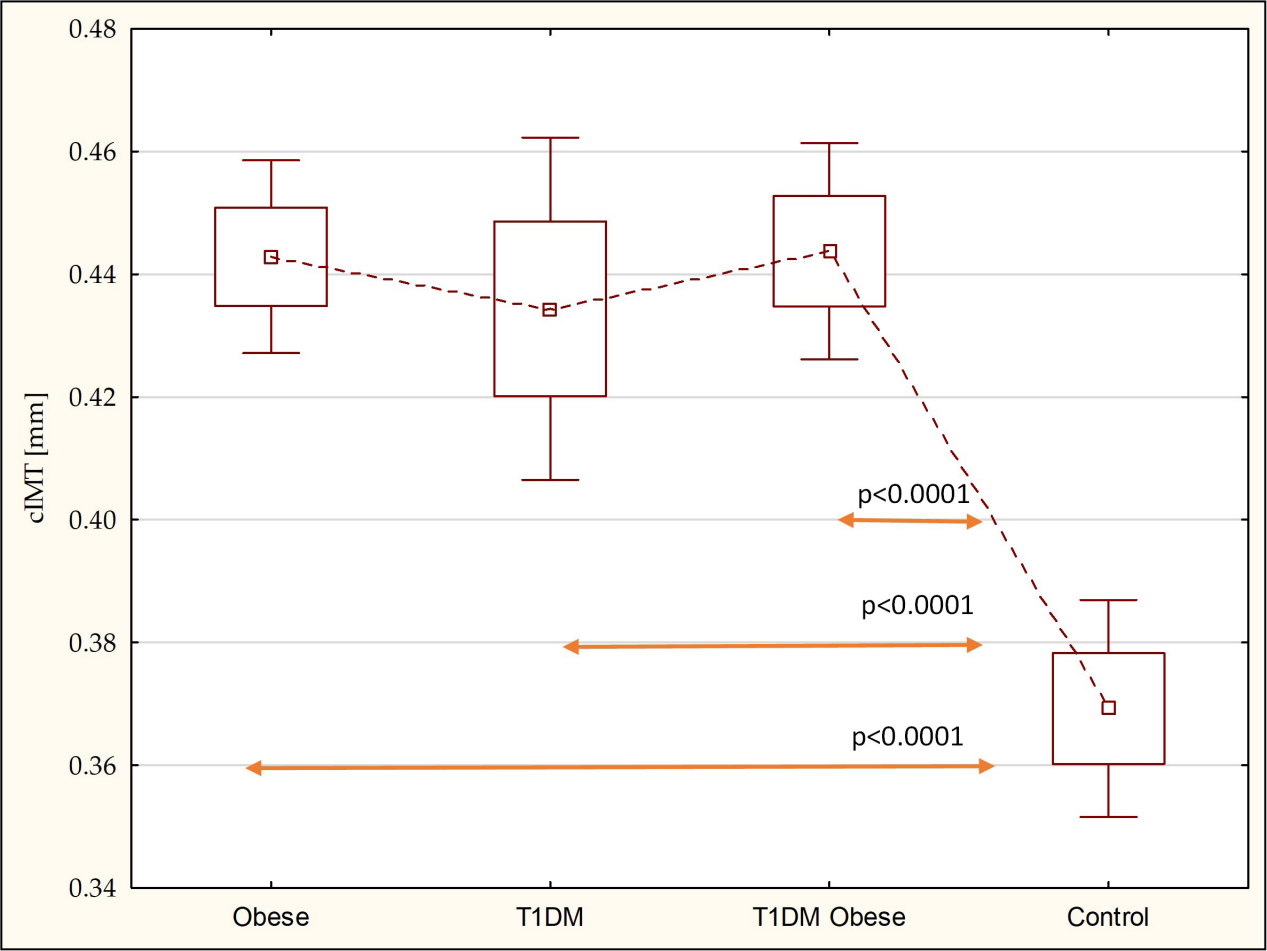
Comparison of the cIMT in the study groups.

There were no statistical differences in intima-media thickness between patients with T1DM with normal weight, 0.47 ± 0.12 [mm], excessive weight, 0.44 ± 0.04 [mm], and non-diabetic obese children, 0.44 ± 0.05 [mm]; however, the cIMT value was higher than the reference group, 0.38 ± 0.03 [mm] (*p* < 0.0001; *p* < 0.0001; *p* < 0.0001) (**Figure 2**).

A significant positive correlation was found among the obese non-diabetic patients between BMI, BMI-SDS, and waist circumference with OPG: *r* = 0.560 (*p* < 0.001); *r* = 0.618 (*p* < 0.001); *r* = 0.547 (*p* < 0.001). Among obese T1D children, fetuin-A levels were significantly positively correlated with BMI, *r* = 0.572 (*p* < 0.001), BMI-SDS, *r* = 0.723 (*p* < 0.0001), HDL [mg/dl], *r* = 0.514 (*p* < 0.01), and daily insulin requirement, *r* = 0.577 (*p* < 0.001), and negatively with eGDR2, *r* = −0.521 (*p* < 0.01).

In non-diabetic obese patients, cIMT was positively correlated with BMI, *r* = 0.675 (*p* = 0.003), BMI-SDS, *r* = 0.679 (*p* = 0.003), waist circumference SDS, *r* = 0.638 (*p* = 0.006), SBP, *r* = 0.6523 (*p* = 0.036), DBP, *r* = 0.600 (*p* = 0.011), TG, *r* = 0.5069 (*p* = 0.017), fasting insulin level, *r* = 0.767 (*p* = 0.000), HOMA-IR, *r* = 0.768 (*p* = 0.000), and OPG level, *r* = 0.528 (*p* = 0.029).

In the group of obese children with T1D, cIMT was positively correlated with TG, *r* = 0.611 (*p* = 0.027), and VAI, *r* = 0.611 (*p* = 0.027). Negative correlation was reported with HDL, *r* = −0.694 (*p* = 0.008).

## Discussion

The relationship of body weight and adiposity with cardiovascular risk factors and novel markers for metabolic complications in children and young adults with T1DM is not fully understood (32).

In the DCCT/EDIC study, excess weight gain among the patients with T1DM was reported to be associated with sustained increases in central obesity, insulin resistance, dyslipidemia, and hypertension, as well as more extensive atherosclerosis (33, 34).

It is important to remember that the American Heart Association classified T1DM as a high cardiovascular risk factor for pediatric patients who are at risk of obesity (35).

Because children and young adults with T1DM and excessive body weight have a higher likelihood of having coexisting hypertension, dyslipidemia, and elevated alanine aminotransferase, the problem of metabolic syndrome among them should also be considered.

Merger et al., in their cross-sectional study, suggest that T1DM with metabolic syndrome is an independent risk factor for T1DM patients in developing macrovascular and microvascular comorbidities (36). Moreover, atherogenic lipid profile is also associated with unsatisfactory diabetes control. The prospective SEARCH study showed that the frequency of dyslipidemia with inadequate glycemic control (HbA1c ≥ 9%), longer T1D duration, obesity, and hypertension correlated with higher cIMT (37). The results from our study showed the association of BMI and BMI-SDS with atherogenic lipid profile and higher blood pressure in obese patients with T1DM, which is consistent with previous studies. However, no significant differences in SBP, DBP, total cholesterol, LDL, and TG levels were found between obese diabetic and non-diabetic patients, suggesting that obesity and its complications, such as diabetes mellitus, might increase the risk of cardiovascular diseases. Relatively little is known about novel markers such as fetuin-A, E-selectin, and OPG among young patients with T1DM and their correlation with body weight, insulin resistance, and risk of cardiovascular diseases. Siraz et al., in their study, presented that patients with fetuin-A levels above the cutoff value had poorer glycemic control and higher TG levels (38). The association between insulin resistance and higher fetuin-A level among the male patients with T1DM was reported in a previous study (39). Moreover, the connection between serum fetuin-A concentration and the development of cardiovascular complications in diabetics has been reported (40).

One of the most intriguing novel findings of our study is that fetuin-A levels were higher among obese non-diabetic children than in obese T1DM. It could be explained by the hypothesis that in patients without diabetes, fetuin-A plays a potentially protective role against coronary artery disease and acute cardiovascular events and also prevents spontaneous mineral precipitation in the vasculature (41–43). This might suggest that in diabetic patients, the inflammatory process occurs faster compared to non-diabetic obese patients, regardless of obesity. Moreover, fetuin-A levels among obese diabetic patients correlated with obesity, higher daily insulin requirement, and insulin resistance. These findings indicate that fetuin-A can be used as a parameter for predicting cardiovascular complications of T1DM and for monitoring poor glycemic control.

However, further research should be performed to establish precise cutoff points.

It is now recognized that a higher E-selectin level is a marker for development of atherosclerosis. Several studies reported increased levels in patients with obesity, metabolic syndrome, and T1DM. It was also noted that among the diabetic patients, there is a positive correlation between HbA1c, diastolic blood pressure, cholesterol, TG, and E-selectin levels.

In our study, serum E-selectin levels were similar among obese patients with T1DM and non-diabetic patients, but higher than the other groups. In contrast to other groups, no obvious correlation between classical risk factors and novel markers was noted (44, 45).

OPG is a bone-related peptide that can be found in different tissues, including bone, heart, and vascular endothelial cells. Current data suggest that increased plasma OPG concentrations are associated with atherosclerosis in the general population and are an independent predictor of cardiovascular complications in a large cohort of patients with T1DM 1 (46).

Our investigations show that OPG levels did not differ between obese T1DM patients and non-diabetic obese subjects, but were higher in healthy individuals. Those findings are in line with those obtained by Gelluzi et al. and Ayina et al. (47). In the study, we also confirmed a positive correlation between OPG and BMI, BMI-SDS, and waist circumference in non-diabetic obese patients. The discrepancy in results may be a consequence of the study population; patients with T1DM and obesity had statistically lower BMI. Moreover, obesity is associated with elevated triglycerides and LDL and low HDL. However, in the present study, OPG levels did not correlate with TG, HDL, or LDL in both groups. Gannage-Yared et al. have detected a positive correlation between OPG and the HOMA index (48). Kim et al. have also determined an association between serum OPG levels and HOMA-IR in both normal and diabetic patients (49). In our study, we could not establish such a correlation between OPG levels and indexes of insulin sensitivity and insulin resistance. The role of OPG in the pathogenesis of atherosclerosis is still unclear. There is a discussion whether OPG synthesis is a compensatory mechanism to counteract the atherosclerotic process or whether OPG is an active compound in the atherosclerotic process (50, 51). Our results suggest that OPG might be involved in metabolic processes associated with atherosclerosis development. Further prospective studies are required to establish whether increased OPG levels in diabetic children in general as well as among those with obesity can predict later development of endothelial dysfunction and vascular complications.

In the study, we also reported that both of our obese groups—the non-diabetic group and the group with T1DM—had significantly higher cIMT compared to the control group. Even though there was no statistical difference between them, differences in cardiovascular risk factors were noticed.

In our non-diabetic obese patients, cIMT was positively correlated with BMI, BMI-SDS, and waist circumference-SDS. These findings are consistent with the data presented by the International Childhood Vascular Structure Evaluation Consortium. Abnormal cIMT was described in patients with cardiovascular complications including hypertension. Even though none of the study subjects was diagnosed with hypertension, there was a strong positive correlation between SBP, DBP, and cIMT among the non-diabetic group (23, 52). Moreover, obesity-related insulin resistance can induce atherothrombotic mechanisms, reduce fibrinolytic balance, and impair endothelial function. The possible relationship between cIMT and insulin resistance/hyperinsulinemia in children and adolescents remains inconsistent, with some studies reporting an adverse relation between insulin resistance and vascular measures while others observed no significant relation at all (53, 54). Our findings are in agreement with results of recent investigations where insulin resistance was associated with carotid wall thickness among non-diabetic children and adolescents with obesity.

Some publications report that T1DM patients have significantly increased cIMT levels compared to control subjects (55, 56). The SEARCH CVD Study clearly stated that increased BMI was a CV risk factor in young people with T1D and influenced cIMT (38).

Data obtained from our study were inconsistent. Similar to other studies, cIMT values were higher among diabetic patients, regardless of BMI, but strongly correlated with visceral adipose tissue, whose activity was expressed by VAI and triglyceride levels. Morisawa et al. indicate that OPG is also significantly associated with endothelial function and its concentration can be a useful predictor of early carotid atherosclerosis and higher cIMT (57). A similar correlation was noted in the group of obese non-diabetic patients.

### Limitations of the study

We realize that our study has some limitations. First of all, the sample size was small, and the small number of patients were included. The age range included children and adults; however, only 10 subjects were older than 18 years (6 in the group with T1DM and normal weight and 4 in the group with T1DM and excessive body weight).

There were differences between the degree of obesity among diabetic and non-diabetic patients, and the stage of puberty was not considered. Further studies are necessary to report the degree of obesity among T1DM children and young adults and its influence on the levels of novel markers as well as cardiovascular complications.

## Conclusion

Obesity in children and young adults with T1DM results in worse metabolic control, insulin resistance, and increased risk for vascular complications. However, novel markers of metabolic complications of obesity are similar between obese diabetic and non-diabetic patients.

## Data availability statement

The original contributions presented in the study are included in the article/supplementary material. Further inquiries can be directed to the corresponding authors.

## Ethics statement

The studies involving human participants were reviewed and approved by The Bioethics Committee of University of Warmia and Mazury (approval number KB/13/2019). Written informed consent to participate in this study was provided by the participants’ legal guardian/next of kin.

## Author contributions

AK conceptualized and designed the study, collected data, performed statistical analysis, prepared tables and figures, and wrote and edited the manuscript. BG-O conceptualized and designed the study, interpreted the results, and designed and revised the manuscript. AC was involved in the design, conception, and revision of the manuscript. EJ-C read and approved the final version of the manuscript. All authors contributed to the article and approved the version submitted.

## Funding

This research was supported by grants from Medical School of University of Warmia and Mazury in Olsztyn, Poland.

## Conflict of interest

The authors declare that the research was conducted in the absence of any commercial or financial relationships that could be construed as a potential conflict of interest.

## Publisher’s note

All claims expressed in this article are solely those of the authors and do not necessarily represent those of their affiliated organizations, or those of the publisher, the editors and the reviewers. Any product that may be evaluated in this article, or claim that may be made by its manufacturer, is not guaranteed or endorsed by the publisher.

## References

[1] Norris JM Johnson RK Stene LC. Type 1 diabetes-early life origins and changing epidemiology. Lancet Diabetes Endocrinol (2020) 8(3):226–38. doi: 10.1016/S2213-8587(19)30412-7 PMC7332108 31999944

[2] World Health Organization (WHO). Obesity and overweight. Available at: https://www.who.int/news-room/fact-sheets/detail/obesity-and-overweight (Accessed 9 June 2021).

[3] Majcher A Czerwonogrodzka-Senczyna A Kądziela K Rumińska M Pyrżak B. Development of obesity from childhood to adolescents. Pediatr Endocrinol Diabetes Metab (2021) 27(2):70–5. doi: 10.5114/pedm.2021.105297 PMC10214958 33860660

[4] Sahoo K Sahoo B Choudhury AK Sofi NY Kumar R Bhadoria AS. Childhood obesity: causes and consequences. J Family Med Prim Care (2015) 4(2):187–92. doi: 10.4103/2249-4863.154628 PMC4408699 25949965

[5] Magliano DJ Boyko EJ. IDF Diabetes Atlas 10th edition scientific committee.. In: IDF DIABETES ATLAS [Internet]. 10th ed.. Brussels: International Diabetes Federation (2021). Available at: https://diabetesatlas.org. 35914061

[6] Ogle GD James S Dabelea D Pihoker C Svennson J Maniam J. Global estimates of incidence of type 1 diabetes in children and adolescents: Results from the international diabetes federation atlas, 10. Diabetes Res Clin Pract (2021) 183:109083. doi: 10.1016/j.diabres.2021.109083 34883188

[7] Manyanga T Sellers EA Wicklow BA Doupe M Fransoo R. Not as skinny as we used to think: Body mass index in children and adolescents at diagnosis of type 1 diabetes mellitus. J Diabetes Complications (2016) 30(2):292–4. doi: 10.1016/j.jdiacomp.2015.11.022 26718935

[8] Jarosz-Chobot P Polanska J Szadkowska A Kretowski A Bandurska-Stankiewicz E Ciechanowska M. Rapid increase in the incidence of type 1 diabetes in polish children from 1989 to 2004, and predictions for 2010 to 2025. Diabetologia (2011) 54(3):508–15. doi: 10.1007/s00125-010-1993-4 PMC3034048 21165594

[9] Wolosowicz M Lukaszuk B Chabowski A. The causes of insulin resistance in type 1 diabetes mellitus: Is there a place for quaternary prevention? Int J Environ Res Public Health (2020) 17(22):8651. doi: 10.3390/ijerph17228651 33233346 PMC7700208

[10] Danielson KK Drum ML. Estrada CL Lipton RB. Racial and ethnic differences in an estimated measure of insulin resistance among individuals with type 1 diabetes. Diabetes Care (2010) 33(3):614–9. doi: 10.2337/dc09-1220 PMC2827519 20007942

[11] Corbin KD Driscoll KA Pratley RE Smith SR Maahs DM Mayer-Davis EJ. Advancing care for type 1 diabetes and obesity network (ACT1ON). obesity in type 1 diabetes: Pathophysiology, clinical impact, and mechanisms. Endocr Rev (2018) 39(5):629–63. doi: 10.1210/er.2017-00191 30060120

[12] Vilarrasa N San Jose P Rubio MÁ Lecube A. Obesity in patients with type 1 diabetes: Links, risks and management challenges. Diabetes Metab Syndr Obes (2021) 14:2807–27. doi: 10.2147/DMSO.S223618 PMC8232956 34188505

[13] Amato MC Giordano C Pitrone M Galluzzo A. Cut-off points of the visceral adiposity index (VAI) identifying a visceral adipose dysfunction associated with cardiometabolic risk in a Caucasian Sicilian population. Lipids Health Dis (2011) 10:183. doi: 10.1186/1476-511X-10-183 22011564 PMC3224548

[14] Pagan LU Gatto M Martinez PF Okoshi K Okoshi MP. Biomarkers in cardiovascular disease: The role of fetuin-a. Arq Bras Cardiol (2022) 118(1):22–3. doi: 10.36660/abc.20210980 PMC8959047 35195204

[15] Shim YS Kang MJ Oh YJ Baek JW Yang S Hwang IT. Fetuin-a as an alternative marker for insulin resistance and cardiovascular risk in prepubertal children. J Atheroscler Thromb (2017) 24(10):1031–8. doi: 10.5551/jat.38323 PMC5656765 28154244

[16] Weikert C Stefan N Schulze MB Pischon T Berger K Joost HG. Plasma fetuin-a levels and the risk of myocardial infarction and ischemic stroke. Circulation (2008) 118(24):2555–62. doi: 10.1161/CIRCULATIONAHA.108.814418 19029462

[17] Tschiderer L Klingenschmid G Nagrani R Willeit J Laukkanen JA Schett G. Osteoprotegerin and cardiovascular events in high-risk populations: Meta-analysis of 19 prospective studies involving 27 450 participants. J Am Heart Assoc (2018) 7(16):e009012. doi: 10.1161/JAHA.118.009012 30369329 PMC6201389

[18] Rashad NM El-Shal AS Shalaby SM Abdel-Nour HM Sarhan WM. Osteoprotegerin expression and serum values in obese women with type 2 diabetes mellitus. Mol Biol Rep (2021) 48(11):7095–104. doi: 10.1007/s11033-021-06699-x PMC8419664 34487291

[19] Kalkan R Becer E. RANK/RANKL/OPG pathway is an important for the epigenetic regulation of obesity. Mol Biol Rep (2019) 46(5):5425–32. doi: 10.1007/s11033-019-04997-z 31364017

[20] Pérez de Ciriza C Moreno M Restituto P Bastarrika G Simón I Colina I. Circulating osteoprotegerin is increased in the metabolic syndrome and associates with subclinical atherosclerosis and coronary arterial calcification. Clin Biochem (2014) 47(18):272–8. doi: 10.1016/j.clinbiochem.2014.09.004 25218813

[21] Al-Shorman A Al-Domi H Faqih A. Markers of subclinical atherosclerosis in schoolchildren with obesity and metabolic syndrome. Swiss Med Wkly (2020) 147:w14446. doi: 10.4414/smw.2020.14446 28634974

[22] Porta B Baldassarre D Camera M Amato M Arquati M Brusoni B. E-selectin and TFPI are associated with carotid intima-media thickness in stable IHD patients: the baseline findings of the MIAMI study. Nutr Metab Cardiovasc Dis (2008) 18(4):320–8. doi: 10.1016/j.numecd.2007.01.008 17889518

[23] Zhao M López-Bermejo A Caserta CA Medeiros CCM Kollias A Bassols J. International childhood vascular structure evaluation consortium. metabolically healthy obesity and high carotid intima-media thickness in children and adolescents: International childhood vascular structure evaluation consortium. Diabetes Care (2019) 42(1):119–25. doi: 10.2337/dc18-1536 30420475

[24] Klonowska B Charemska D Jabłońska J Banach A Kącka A Szynkarczuk E. Carotid artery intima-media thickness (cIMT) in young type 1 diabetic patients in relation to comorbid additional autoimmune diseases and microvascular complications. Pediatr Endocrinol Diabetes Metab (2016) 22(3). doi: 10.18544/PEDM-22.03.0057 28633159

[25] Sun YP Cai YY Li HM Deng SM Leng RX Pan HF. Increased carotid intima-media thickness (CIMT) levels in patients with type 1 diabetes mellitus (T1DM): A meta-analysis. J Diabetes Complications (2015) 29(5):724–30. doi: 10.1016/j.jdiacomp.2015.03.018 25890843

[26] . Available at: https://www.mp.pl/pediatria/praktyka-kliniczna/procedury/13874,rys-11-siatki-centylowe-wskaznika-wzglednej-masy-ciala-bmi-chlopcow-a-i-dziewczat-b-warszawskich.

[27] Majid H Masood Q Khan AH. Homeostatic model assessment for insulin resistance (HOMA-IR): A better marker for evaluating insulin resistance than fasting insulin in women with polycystic ovarian syndrome. J Coll Phys Surg Pak (2017) 27(3):123–6. 28406767

[28] Guidelines on the management of patients with diabetes 2021. Available at: https://ptdiab.pl/images/docs/zalecenia/2021-Guidelines-on-the-management-of-patients-with-diabetes.pdf.

[29] Williams KV Erbey JR Becker D Arslanian S Orchard TJ. Can clinical factors estimate insulin resistance in type 1 diabetes? Diabetes (2000) 49(4):626–32. doi: 10.2337/diabetes.49.4.626 10871201

[30] Szadkowska A Pietrzak I Szlawska J Kozera A Gadzicka A Młynarski W. Abdominal obesity, metabolic syndrome in type 1 diabetic children and adolescents. Pediatr Endocrinol Diabetes Metab (2009) 15(4):233–9. Available at: https://www.researchgate.net/publication/44585398_Abdominal_obesity_metabolic_syndrome_in_type_1_diabetic_children_and_adolescents. 20455417

[31] Pignoli P Tremoli E Poli A Oreste P Paoletti R. Intimal plus medial thickness of the arterial wall: a direct measurement with ultrasound imaging. Circulation (1986) 74(6):1399–406. doi: 10.1161/01.cir.74.6.1399 3536154

[32] Salgado AL Carvalho LD Oliveira AC Santos VN Vieira JG Parise ER. Insulin resistance index (HOMA-IR) in the differentiation of patients with non-alcoholic fatty liver disease and healthy individuals. Arq Gastroenterol (2010) 47(2):165–9. doi: 10.1590/s0004-28032010000200009 20721461

[33] Jamiołkowska-Sztabkowska M Głowińska-Olszewska B Bossowski A. C-peptide and residual β-cell function in pediatric diabetes – state of the art. Pediatr Endocrinol Diabetes Metab (2021) 27(2):123–33. doi: 10.5114/pedm.2021.107165 PMC10214969 34514768

[34] Lipsky LM Gee B Liu A Nansel TR. Body mass index and adiposity indicators associated with cardiovascular biomarkers in youth with type 1 diabetes followed prospectively. Pediatr Obes (2017) 12(6):468–76. doi: 10.1111/ijpo.12167 PMC8211376 27417272

[35] Purnell JQ Zinman B Brunzell JD DCCT/EDIC Research Group. The effect of excess weight gain with intensive diabetes mellitus treatment on cardiovascular disease risk factors and atherosclerosis in type 1 diabetes mellitus: results from the diabetes control and complications Trial/Epidemiology of diabetes interventions and complications study (DCCT/EDIC) study. Circulation (2013) 127(2):180–7. doi: 10.1161/CIRCULATIONAHA.111.077487 PMC3819101 23212717

[36] de Ferranti SD Steinberger J Ameduri R Baker A Gooding H Kelly AS. Cardiovascular risk reduction in high-risk pediatric patients: A scientific statement from the American heart association. Circulation (2019) 139(13):e603–34. doi: 10.1161/CIR.0000000000000618 30798614

[37] Merger SR Kerner W Stadler M Zeyfang A Jehle P Müller-Korbsch M. DPV initiative; German BMBF competence network diabetes mellitus. prevalence and comorbidities of double diabetes. . Diabetes Res Clin Pract (2016) 119:48–56. doi: 10.1016/j.diabres.2016.06.003 27449710

[38] Shah AS Dabelea D Fino NF Dolan LM Wadwa RP D'Agostino R Jr. Predictors of increased carotid intima-media thickness in youth with type 1 diabetes: The SEARCH CVD study. Diabetes Care (2016) 39(3):418–25. doi: 10.2337/dc15-1963 PMC4764035 26721813

[39] Şiraz ÜG Doğan M Hatipoğlu N Muhtaroğlu S Kurtoğlu S. Can fetuin-a be a marker for insulin resistance and poor glycemic control in children with type 1 diabetes mellitus? J Clin Res Pediatr Endocrinol (2017) 9(4):293–9. doi: 10.4274/jcrpe.4532 PMC5785634 28529199

[40] Jung CH Kim BY Kim CH Kang SK Jung SH Mok JO. Associations of serum fetuin-a levels with insulin resistance and vascular complications in patients with type 2 diabetes. Diabetes Vasc Dis Res (2013) 10(5):459–67. doi: 10.1177/1479164113490766 23811603

[41] Dabrowska AM Tarach JS Wojtysiak-Duma B Duma D. Fetuin-a (AHSG) and its usefulness in clinical practice. review of the literature. BioMed Pap Med Fac Univ Palacky Olomouc Czech Repub (2015) 159(3):352–9. doi: 10.5507/bp.2015.018 25916279

[42] Vörös K Gráf L Jr Prohászka Z Gráf L Szenthe P Kaszás E. Serum fetuin-a in metabolic and inflammatory pathways in patients with myocardial infarction. Eur J Clin Invest (2011) 41(7):703–9. doi: 10.1111/j.1365-2362.2010.02456.x 21226708

[43] Zhao ZW Lin CG Wu LZ Luo YK Fan L Dong XF. Serum fetuin-a levels are associated with the presence and severity of coronary artery disease in patients with type 2 diabetes. Biomarkers (2013) 18(2):160–4. doi: 10.3109/1354750X.2012.762806 23410047

[44] Carrizo Tdel R Prado MM Velarde MS Díaz EI Bazán MC Abregú AV. E-selectina soluble en una población infanto-juvenil con diabetes tipo 1 [Soluble e- selectin in children and adolescents with type 1 diabetes. Med (B Aires) (2008) 68(3):193–7. Available at: https://www.researchgate.net/publication/23157909_Soluble_E-selectin_in_children_and_adolescents_with_type_1_diabetes. 18689149

[45] El Wakeel MA El-Kassas GM Amer AF Elbatal WH Sabry RN EL-Ghaffar M. E-selectin and vascular complications in children with type 1 diabetes mellitus. Med Res J (2014) 13(1):27–32. doi: 10.1097/01.MJX.0000446937.40653.3d

[46] Galluzzi F Stagi S Salti R Toni S Piscitelli E Simonini G. Osteoprotegerin serum levels in children with type 1 diabetes: a potential modulating role in bone status. Eur J Endocrinol (2005) 153(6):879–85. doi: 10.1530/eje.1.02052 16322394

[47] Ayina Ayina CN Sobngwi E Essouma M Noubiap JJN Boudou PH Ngoa LSE. Osteoprotegerin in relation to insulin resistance and blood lipids in sub-Saharan African women with and without abdominal obesity. Diabetol Metab Syndr (2015) 7:47. doi: 10.1186/s13098-015-0042-3 26034511 PMC4450452

[48] Gannagé-Yared MH Yaghi C Habre B Khalife S Noun R Germanos-Haddad M. Osteoprotegerin in relation to body weight, lipid parameters insulin sensitivity, adipocytokines, and c-reactive protein in obese and non-obese young individuals: results from both cross-sectional and interventional study. Eur J Endocrinol (2008) 158(3):353–9. doi: 10.1530/EJE-07-0797 18299469

[49] Yeşilkaya E Bideci A Çamurdan O Boyraz M Vurucu S Cinaz P. Association of osteoprotegerin and rankl levels with insulin resistance in pubertal obese children. Open Med (2009) 5:261–7. doi: 10.2478/s11536-009-0065-y

[50] Gordin D Soro-Paavonen A Thomas MC. Osteoprotegerin is an independent predictor of vascular events in Finnish adults with type 1 diabetes. Diabetes Care (2013) 36:1827–33. doi: 10.2337/dc12-2170 PMC3687299 23801795

[51] Lambrinoudaki I Tsouvalas E Vakaki M Kaparos G Stamatelopoulos K Augoulea A. Osteoprotegerin, soluble receptor activator of nuclear factor- κ b ligand, and subclinical atherosclerosis in children and adolescents with type 1 diabetes mellitus. Int J Endocrinol (2013) 2013:102120. doi: 10.1155/2013/102120 24288529 PMC3833004

[52] Widjaja NA Irawan R Prihaningtyas RA Ardiana M Hanindita MH. Carotid intima-media thickness, hypertension, and dyslipidemia in obese adolescents. Pan Afr Med J (2019) 34:134. doi: 10.11604/pamj.2019.34.134.18309 33708303 PMC7906559

[53] Santos IS Bittencourt MS Goulart AC Schmidt MI Diniz MFHS Lotufo PA. Insulin resistance is associated with carotid intima-media thickness in non-diabetic subjects. a cross-sectional analysis of the ELSA-brasil cohort baseline. Atherosclerosis (2017) 260:34–40. doi: 10.1016/j.atherosclerosis.2017.03.011 28340367

[54] Asghari G Dehghan P Mirmiran P Yuzbashian E Mahdavi M Tohidi M. Insulin metabolism markers are predictors of subclinical atherosclerosis among overweight and obese children and adolescents. BMC Pediatr (2018) 18(1):368. doi: 10.1186/s12887-018-1347-9 30470212 PMC6260656

[55] Cerit MN Sendur HN Bolayır B Cerit ET Cindil E Yaşım Aktürk M. Evaluation of common carotid artery in type 1 diabetes mellitus patients through speckle tracking carotid strain ultrasonography. Diagn Interv Radiol (2021) 27(2):195–205. doi: 10.5152/dir.2021.20025 33599210 PMC7963368

[56] Stanković SM Zivić SR Šaranac L Cvetković V Pešić M Vasić K. Determinants of atherosclerosis in children and adolescents with diabetes type 1. Endokrynol Pol (2012) 63(6):414–9. Available at: https://journals.viamedica.pl/endokrynologia_polska/article/view/25127/19956. 23338997

[57] Morisawa T Nakagomi A Kohashi K Kosugi M Kusama Y Atarashi H. Osteoprotegerin is associated with endothelial function and predicts early carotid atherosclerosis in patients with coronary artery disease. Int Heart J (2015) 56(6):605–12. doi: 10.1536/ihj.15-150 26549398

